# Dietary Macronutrient Composition Is Associated with Distinct Hepatic Phenotypes During Post-Weaning Growth: An Integrated Biochemical, Histological, Immunohistochemical and Ultrastructural Study

**DOI:** 10.3390/metabo16100781

**Published:** 2026-10-15

**Authors:** Emrah Zayman, Elif Kayhan, Mehmet Erman Erdemli, Zeynep Erdemli, Feyza İnceoğlu, Mehmet Gül

**Affiliations:** 1 Department of Histology and Embryology, Faculty of Medicine, Malatya Turgut Özal University, Malatya 44210, Türkiye; elif.kayhan@ozal.edu.tr; 2 Department of Medical Biochemistry, Faculty of Medicine, Inonu University, Malatya 44280, Türkiye; erman.erdemli@inonu.edu.tr (M.E.E.); zeynepaksungur.44@gmail.com (Z.E.); 3 Department of Biostatistics and Medical Informatics, Faculty of Medicine, Malatya Turgut Özal University, Malatya 44210, Türkiye; feyza.inceoglu@ozal.edu.tr; 4 Department of Histology and Embryology, Faculty of Medicine, Inonu University, Malatya 44280, Türkiye

**Keywords:** dietary patterns, oxidative stress, liver injury, inflammation, post-weaning development

## Abstract

**Background/Objectives:** The aim of this study was to compare the biochemical, histological, immunohistochemical and ultrastructural hepatic phenotypes associated with carbohydrate-, fat- and protein-enriched diets initiated during post-weaning growth. **Methods:** Forty 21-day-old male Wistar albino rats were divided into four groups: a control group and three experimental groups (carbohydrate, protein, and fat). The respective diets were fed for 13 weeks. Hepatic oxidant–antioxidant parameters, serum aspartate aminotransferase (AST), alanine aminotransferase (ALT), and alkaline phosphatase (ALP), histopathological scores; interleukin (IL)-6 and tumor necrosis factor alpha (TNF-α) immunoreactivity and transmission electron microscopy (TEM) findings were evaluated. **Results:** The carbohydrate group showed higher thiobarbituric acid-reactive substances (TBARS), total oxidant status (TOS) and oxidative stress index (OSI), together with lower reduced glutathione (GSH) and total antioxidant status (TAS). Histology showed hydropic change, vacuolization and inflammatory cell infiltration, while TEM showed mitochondrial swelling, loss of cristae and nuclear alterations. The fat group showed prominent vacuolization, TEM-observed lipid droplets, inflammatory cell infiltration and TNF-α immunoreactivity. Most findings in the protein group were closer to control values. Thus, the carbohydrate-enriched diet was associated with the most pronounced redox and ultrastructural injury profile, whereas the fat-enriched diet was associated with a morphological and inflammatory injury profile compatible with lipid accumulation. **Conclusions:** These findings identify distinct hepatic phenotypes associated with the dietary formulations administered during post-weaning growth. The carbohydrate- and fat-enriched formulations were associated with more pronounced biochemical and morphological changes; inference is made at the level of the complete dietary formulation.

## 1. Introduction

Nutrition is fundamental to post-natal growth, development, tissue integrity and metabolic homeostasis [1,2]. Dietary nutrients provide energy and contribute to redox balance, inflammatory responses, cellular repair, and organ function [3,4]. Unhealthy dietary patterns can disrupt oxidant–antioxidant balance and promote chronic metabolic inflammation [5,6]. Therefore, nutritional effects should be evaluated not only through calorie intake and body weight change but also through histological, biochemical, and tissue-level responses of target organs [7,8].

Carbohydrates, fats, and proteins are the fundamental macronutrients in the diet and key determinants of the metabolic outcomes of dietary patterns [7,9]. Carbohydrates are primarily associated with glucose metabolism and glycogen storage as a rapid energy source. A high carbohydrate intake significantly affects hepatic lipogenesis, insulin signaling, and glycogen metabolism [10]. In addition to their high energy density, fats are important for membrane structure, cellular signaling, and lipid transport. However, high-fat diets are associated with hepatic lipid accumulation, oxidative stress, and models of non-alcoholic fatty liver disease [6,11]. Protein intake is important for nitrogen metabolism and tissue protein turnover. However, the impact of high-protein diets on the liver depends on factors such as the duration of the diet, the protein ratio, and accompanying metabolic conditions [12,13].

The liver performs numerous vital functions at the intersection of carbohydrate, lipid and protein metabolism. These include glycogen synthesis and degradation, lipogenesis, beta-oxidation, amino acid metabolism, detoxification, and plasma protein synthesis [3,7]. This intense metabolic activity makes the liver susceptible to reactive oxygen species, inflammatory mediators, and metabolic overload induced by nutrients [3,4]. Oxidative stress is known to lead to lipid, protein, and DNA damage in hepatocytes, and the liver is more sensitive to chronic oxidative conditions than many other organs [3,4]. Consequently, the macronutrient composition of the diet is expected to significantly impact the histological architecture of hepatocytes, the inflammatory cell response, cytokine expression, and antioxidant defense systems [5,7].

Postnatal liver maturation is not simply a scaled version of adult liver physiology. In rodents, the transition from suckling to solid feeding is accompanied by coordinated changes in ingestive behavior, digestion, and hepatic metabolism [1]. Experimental evidence in mice further shows that hepatic immune cell composition and macronutrient-metabolizing functions continue to reorganize during the neonatal-to-weaning transition and that premature weaning can disturb the expression of hepatic metabolic pathways [14]. In mice, postnatal liver growth also follows a stage-dependent relationship with somatic growth, with the liver-to-body weight ratio changing as the organ approaches its adult proportion [15]. These observations indicate that a diet introduced at weaning is delivered while both whole-body growth and hepatic structural and functional maturation are still ongoing.

Although the hepatic effects of high-fat, high-carbohydrate, and high-protein diets have each been investigated [3,8,9,12,16,17,18], the present study does not seek to establish that these individual dietary effects are previously unknown. Rather, it addresses a comparative question by evaluating different macronutrient-enriched diets within the same experimental design. By applying identical experimental conditions (same strain, sex, age at diet initiation, duration of dietary exposure, and housing conditions) together with integrated biochemical, histopathological, immunohistochemical, and ultrastructural assessments, the study aims to provide a standardized comparison of the hepatic phenotypes associated with different macronutrient compositions. Evidence that the starch and fiber contents of purified control diets can influence hepatic lipid homeostasis further supports considering the complete formulation when interpreting dietary comparisons [19].

Against this background, the starting age of 21 days is a biologically important design feature. Rats at this age are newly weaned and still in a rapid prepubertal growth phase; age and body weight can materially influence gene expression, nutrient and drug metabolism, and other experimental endpoints [20]. Thus, the present intervention evaluates how different macronutrient-enriched diets shape hepatic phenotypes across the transition from weaning through maturation. Its principal novelty is therefore developmental and comparative: it examines the growing organism and developing liver under standardized dietary exposures, rather than simply reproducing established diet-induced liver injury in adult animals.

Accordingly, the aim of this study was to compare the hepatic phenotypes associated with carbohydrate-, fat-, and protein-enriched diets initiated immediately after weaning in 21-day-old male Wistar albino rats and maintained for 13 weeks under identical experimental conditions. In addition to monitoring growth-related parameters throughout the experimental period, hepatic oxidant–antioxidant balance, serum liver enzymes, histopathological damage scores, interleukin (IL)-6 and tumor necrosis factor alpha (TNF-α) immunohistochemical expression, and hepatocyte ultrastructure were evaluated to characterize the predominant biochemical and morphological hepatic phenotypes associated with different macronutrient patterns during post-weaning development. Thus, the intended contribution of this study is an integrated comparison of nutritional effects on the developing liver rather than the identification of a novel molecular mechanism.

## 2. Materials and Methods

### 2.1. Animals

The study was conducted in accordance with national and international guidelines for animal welfare and approved by the Animal Experimentation Ethics Committee of Inonu University (protocol code: 2019/15-1, date of approval: 12 December 2019). All procedures involving the subjects were carried out at the Inonu University Experimental Animals Research and Production Center in accordance with the ethics committee protocol. Forty 21-day-old male Wistar albino rats, each weighing 35–40 g, were divided into four groups of ten rats. The rats were housed in a well-ventilated room under a 12 h light/dark cycle, with a constant room temperature and relative humidity, throughout the experiment. Sample-size planning was evaluated in G*Power 3.1.9.4 using its ‘A priori: compute required sample size’ setting for a fixed-effects, one-way, four-group omnibus ANOVA. With an assumed effect size f = 1.0365417, α = 0.05, and target power (1 − β) = 0.95, the software returned a minimum total sample of 24 animals (six per group; calculated power 0.9809671 at *N* = 24). Ten animals per group (*N* = 40) were included to provide a margin for possible sample loss and preserve analyzable group sizes [21,22]. No animals were excluded from the experiment, and all 40 animals remained in their assigned groups throughout the study.

1. Control group (*n* = 10): fed a purified control diet detailed in Supplementary Table S1.
2. Carbohydrate group (*n* = 10): fed a purified experimental diet containing 70 kcal% carbohydrate.
3. Fat group (*n* = 10): fed a purified experimental diet containing 60 kcal% fat.
4. Protein group (*n* = 10): fed a purified experimental diet containing 50 kcal% protein.

The 21-day-old rats were placed in cages of five and fed special experimental diets (Research Diets, Inc., 20 Jules Lane, New Brunswick, NJ, USA) for 13 weeks (see Supplementary Table S1) [23]. The experimental diets were purified custom formulations manufactured by Research Diets, Inc., and the same macronutrient formulations were previously reported in post-weaning female Wistar rats [23]. Macronutrient composition was expressed as a percentage of total energy. The percentages of energy derived from protein, carbohydrate, and fat were, respectively, 24%, 64%, and 12% in the control group; 18%, 70%, and 12% in the carbohydrate group; 20%, 20%, and 60% in the fat group; and 50%, 38%, and 12% in the protein group. The control and carbohydrate formulations had similar compositions, with the principal change being an increase in corn starch accompanied by a decrease in casein. The control and carbohydrate-enriched formulations were energy-matched at 4057 kcal per reported formulation and 3.9 kcal/g, with fat held at 12 kcal%. Within this pair, carbohydrate replaced protein on an equal-energy basis: carbohydrate increased from 64 to 70 kcal% (+6 percentage points), whereas protein decreased from 24 to 18 kcal% (−6 percentage points). At the ingredient level, 60.6 g of additional corn starch (approximately 242 kcal) replaced 60.8 g of casein (approximately 243 kcal), while maltodextrin, sucrose, soybean oil, lard, cellulose, and the listed micronutrient ingredients were unchanged. Accordingly, the carbohydrate group represents an isocaloric carbohydrate-for-protein substitution formulation and was not designed to estimate an isolated carbohydrate main effect at fixed protein. Equal energy density does not imply equal energy intake per animal; the unit of inference in these comparisons is the administered dietary formulation. The fat-enriched diet provided 5.2 kcal/g, whereas the control, carbohydrate-enriched, and protein-enriched diets provided 3.9 kcal/g; all diets were offered ad libitum (Supplementary Table S1).

### 2.2. Tissue Preparation and Assessment of Serum Aspartate Aminotransferase (AST), Alanine Aminotransferase (ALT) and Alkaline Phosphatase (ALP) Levels

At the end of the experimental period, the animals were anesthetized by intraperitoneal administration of ketamine (90 mg/kg) and xylazine (9 mg/kg). Animals were not fasted and had unrestricted access to food and water until anesthesia induction. Blood was collected into collection tubes immediately after anesthesia induction, and serum was subsequently separated by centrifugation. Blood samples were handled promptly and carefully to minimize the risk of hemolysis. The same anesthesia and blood-sampling procedures were applied across all experimental groups. Serum ALT (OSR6107, Beckman Coulter Inc. Brea, CA, USA), AST (OSR6109, Beckman Coulter Inc. Brea, CA, USA) and ALP (OSR6104, Beckman Coulter Inc. Brea, CA, USA) levels were measured using an Abbott Architect c16000 analyzer (Abbott Diagnostics, Abbott Park, IL, USA). The livers were excised immediately and gently rinsed with phosphate-buffered saline (PBS) to remove visible residual blood. Aliquots allocated for biochemical analysis were blotted dry, weighed, and homogenized in PBS at a 1:9 (*w*/*v*) tissue-to-PBS ratio for subsequent analyses. A portion of each liver was placed in a sterile container and stored immediately at −80 °C for biochemical analysis. For antioxidant enzyme assays, liver tissues were homogenized and centrifuged, and the resulting supernatants were used for the determination of enzymatic antioxidant parameters. Samples approximately 1 mm^3^ in volume were fixed in 2.5% glutaraldehyde for transmission electron microscopy (TEM), and the remaining tissue was fixed in 10% neutral buffered formalin for histological processing. The weighing step used to establish the homogenization ratio refers to the amount of tissue allocated to analysis; it was not used as a measure of whole-organ growth.

### 2.3. Assessment of Oxidative Stress and Antioxidant Status in Liver Tissue

For biochemical analysis, liver tissue was homogenized in PBS at 4 °C for 60 s at 16,000 rpm using an IKA homogenizer (Staufen, Germany). Thiobarbituric acid-reactive substances (TBARS) were measured directly in the homogenate. The remaining homogenate was centrifuged at 4 °C for 20 min at 600× *g*. Following centrifugation, the supernatant, representing the crude supernatant obtained under these centrifugation conditions, was carefully collected, while the lower pellet containing sedimented erythrocytes and other cellular debris was discarded. Reduced glutathione (GSH), superoxide dismutase (SOD), catalase (CAT), total oxidant status (TOS), and total antioxidant status (TAS) were measured in the resulting supernatant.

#### 2.3.1. TBARS Measurement

The measurement of TBARS was performed according to the method described by Mihara and Uchiyama [24]. Briefly, 0.5 mL of tissue homogenate was mixed with 2 mL of 1% H_3_PO_4_ and 0.5 mL of 0.6% thiobarbituric acid solution. The mixture was heated in a boiling water bath for 45 min and subsequently extracted with 2 mL of n-butanol. n-Butanol was used as the blank, and tetramethoxypropane was used as the standard. The absorbance of the extracted samples was measured using a T80 UV/VIS spectrophotometer (PG Instruments Ltd., Leicestershire, UK) at 535 nm and 520 nm according to the original method. TBARS levels were calculated based on the absorbance values obtained from these measurements and expressed as lipid peroxidation products.

#### 2.3.2. Reduced GSH Measurement

Reduced GSH was determined according to Ellman [25]. The assay is based on spectrophotometric assessment of the reaction with 5,5′-dithiobis(2-nitrobenzoic acid) (DTNB; Ellman’s reagent). DTNB reacts with accessible sulfhydryl groups to form the yellow 5-thio-2-nitrobenzoate (TNB) anion. Absorbance was measured at 410 nm, and GSH concentrations were calculated according to the assay protocol.

#### 2.3.3. Measurement of SOD Activity

SOD activity was determined using the method described by Jolitha et al. [26]. Superoxide radicals were generated using xanthine and xanthine oxidase and reduced nitroblue tetrazolium (NBT) to blue formazan. SOD activity was calculated from the inhibition of formazan formation measured at 560 nm.

#### 2.3.4. Measurement of CAT Activity

CAT activity was determined according to the method described by Aebi [27]. Hydrogen peroxide (H_2_O_2_) exhibits maximum absorbance at 240 nm. Liver tissue homogenates were centrifuged, and CAT activity was measured in the resulting supernatants by monitoring the decrease in absorbance at 240 nm due to the decomposition of H_2_O_2_ into H_2_O and O_2_. The decrease in absorbance was recorded continuously for 1 min, and enzyme activity was calculated as the first-order rate constant (K) according to the following equation: K = 1/Δt1 × ln (E_final_/E_initial_) = 2.3/Δt × log(E_final_/E_initial_), where Δt is the measured reaction time, *E* is the optical density measured at 240 nm, and 2.3 is the conversion factor from the natural logarithm (ln) to the common logarithm (log). CAT activity was expressed as the first-order rate constant normalized to protein content (K/g protein).

#### 2.3.5. Measurement of Total Protein Levels

The total protein content of the supernatant sample was determined using the method reported by Lowry et al. [28]. Protein concentration was measured at 700 nm using a spectrophotometer.

#### 2.3.6. TOS Measurement

TOS was determined using a commercially available Rel Assay Diagnostics kit (Catalog No: RL0024; Rel Assay Diagnostics, Gaziantep, Türkiye) according to the manufacturer’s instructions and the method described by Erel [29]. Briefly, 30 µL of liver tissue supernatant was mixed with 200 µL of Reagent 1, and the initial absorbance was measured at 530 nm. Subsequently, 10 µL of Reagent 2 was added, and the reaction mixture was incubated at 37 °C for 5 min before the absorbance was measured again at 530 nm. The assay is based on the oxidation of ferrous ions to ferric ions by oxidant species present in the sample, and the results are expressed as μmol H_2_O_2_ equivalents/L.

#### 2.3.7. TAS Measurement

TAS was determined using a commercially available Rel Assay Diagnostics kit (Catalog No: RL0017; Rel Assay Diagnostics, Gaziantep, Türkiye) according to the manufacturer’s instructions and the method described by Erel [30]. Briefly, 12 µL of liver tissue supernatant was mixed with 200 µL of Reagent 1, and the initial absorbance was measured at 660 nm. Subsequently, 30 µL of Reagent 2 was added, and the reaction mixture was incubated at 37 °C for 5 min before the absorbance was measured again at 660 nm. The assay is based on the suppression of ABTS•^+^ radical cation formation by antioxidants present in the sample, and the results are expressed as mmol Trolox equivalents/L.

#### 2.3.8. Oxidative Stress İndex (OSI)

OSI was calculated as the ratio of TOS to TAS according to the method described by Erel [29,31]. OSI was calculated using the following formula: OSI (arbitrary units) = TOS (μmol H_2_O_2_ equivalent/L)/TAS (millimoles Trolox equivalent/L) × 10. In this representation, the factor of 10 is an arbitrary scaling coefficient, rather than a physical conversion between µmol and mmol. As a derived index of TOS and TAS, OSI was interpreted alongside the other redox findings.

### 2.4. Histopathological Analysis

The liver tissue samples were fixed in 10% neutral buffered formalin and dehydrated using an ethanol series (70%, 96% and 99.9%). The samples were then dissolved in a xylene series and embedded in paraffin wax. Five-micrometer sections were cut from the paraffin-embedded liver specimens, placed on slides, and stained with hematoxylin and eosin (H&E) to assess histopathological changes, including hydropic change, inflammatory cell infiltration, and hepatocellular vacuolization. Liver damage was scored based on changes in intracytoplasmic hydropic degeneration, inflammatory cell infiltration, and vacuolization. Histopathological changes in the liver sections stained with H&E (intracytoplasmic hydropic changes, inflammatory cell infiltration, and vacuolization) were scored from 0 to 3 (0: absent; 1: mild/rare; 2: moderate; 3: severe/common), with a maximum histopathological score of 9 [32,33].

The immunoreactivity of IL-6 and TNF-α was determined by immunohistochemical staining. Following deparaffinization, heat-induced antigen retrieval was performed in citrate buffer at 122 °C for 16 min. Sections were washed once with distilled water and once with phosphate-buffered saline (PBS). Endogenous peroxidase activity was blocked with hydrogen peroxide for 11 min, followed by a PBS wash. A protein-blocking reagent was applied for 5 min to reduce nonspecific binding. Sections were subsequently incubated overnight with 200 μL of the respective primary antibody solution: anti-IL-6 (sc-28343, 1:200) or anti-TNF-α (sc-52746, 1:200), both obtained from Santa Cruz Biotechnology, Inc., Dallas, TX, USA. Following primary antibody incubation and PBS washing, secondary antibodies were applied. Detection was performed using streptavidin–horseradish peroxidase for 16 min, followed by a PBS wash and development with 3-amino-9-ethylcarbazole (AEC) chromogen for 6 min. Sections were washed with PBS and distilled water, counterstained with hematoxylin for 4 min, and rinsed three times with tap water and once with distilled water. IHC scores represent semi-quantitative tissue immunoreactivity. Predominantly cytoplasmic immunoreactivity is expected for IL-6 and TNF-α. Nuclear staining is not considered an expected target-specific pattern for either cytokine. The source dataset records an ordinal IL-6 and TNF-α immunoreactivity score from 0 to 3 for each rat; stained cell percentages are not part of that dataset. The described cytokine chromogen distribution is distinguished from hematoxylin nuclear counterstaining, and these scores are interpreted as tissue-level staining observations rather than absolute cytokine concentrations [34].

All histopathological and immunohistochemical assessments, including semiquantitative scoring, were performed by a histologist blinded to experimental group allocation using a Nikon Eclipse Ni-U light microscope fitted with a Nikon DS-Fi3 camera and analyzed using a Nikon NIS-Elements Documentation 5.02 image analysis system (Nikon Corporation, Tokyo, Japan). The evaluator was unaware of the dietary group identities during assessment.

### 2.5. TEM Analysis

Approximately 1 mm^3^ tissue samples were taken from the liver and fixed with 2.5% glutaraldehyde at 4 °C for 12 h for electron microscopy examination. Following fixation, tissue tracking procedures were performed using the Leica EM AMW device (Leica Mikrosysteme GmbH, Vienna, Austria). The tissues were embedded in resin in silicone molds and left to polymerize in a preheated oven at 45 °C for 4 h. The temperature was then increased to 60 °C, and polymerization was continued for an additional 24 h. The resin blocks were trimmed using a Leica EM Trimmer (Leica Microsystems, Vienna, Austria), and then 80 nm thick sections were collected on 200 mesh copper grids using an ultramicrotome (Leica Ultracut R) for ultra-thin sections. The sections were contrasted with uranyl acetate and lead citrate, after which they were analyzed using a Zeiss Libra 120 TEM (Carl Zeiss Microscopy, Oberkochen, Germany). Photographs were then taken. TEM was used for descriptive assessment of intracellular structures; representative images were not used to infer quantitative organelle function or morphometric differences between groups. As the images represent selected microscopic fields, they support morphological description but not population-level mitochondrial morphometry; both sampling and preparation conditions affect ultrastructural interpretation [35]. Evaluation of the TEM findings was performed by a histologist blinded to experimental group allocation.

### 2.6. Statistical Analysis

Data were analyzed using SPSS version 26 and Python 3.12.14. Because continuous outcomes did not satisfy the normality assumption, overall group comparisons used the Kruskal–Wallis test, followed by Bonferroni-adjusted Dunn tests for variables with an overall difference. Dunn comparisons were performed for the six group pairs within each outcome. Tie-corrected Kruskal–Wallis tests were applied to biochemical and ordinal histological outcomes, and overall *p* values were obtained using the chi-square approximation. The figures display mean ± standard deviation (SD) with raw data points for descriptive visualization, whereas statistical inference is based on the nonparametric tests. The significance threshold was 0.05. Medians, quartiles, and overall η^2^_H effect sizes are presented with the other descriptive statistics in Supplementary Tables S2–S5. Longitudinal body weights and food intake were assessed descriptively at the group level (Supplementary Tables S6 and S7 and Supplementary Figure S1). The G*Power sample-size calculation used an omnibus parametric F test as a planning benchmark, and the power calculation relates to that planning model.

## 3. Results

### 3.1. Body Weight and Food Intake Patterns

**Figure 1:**
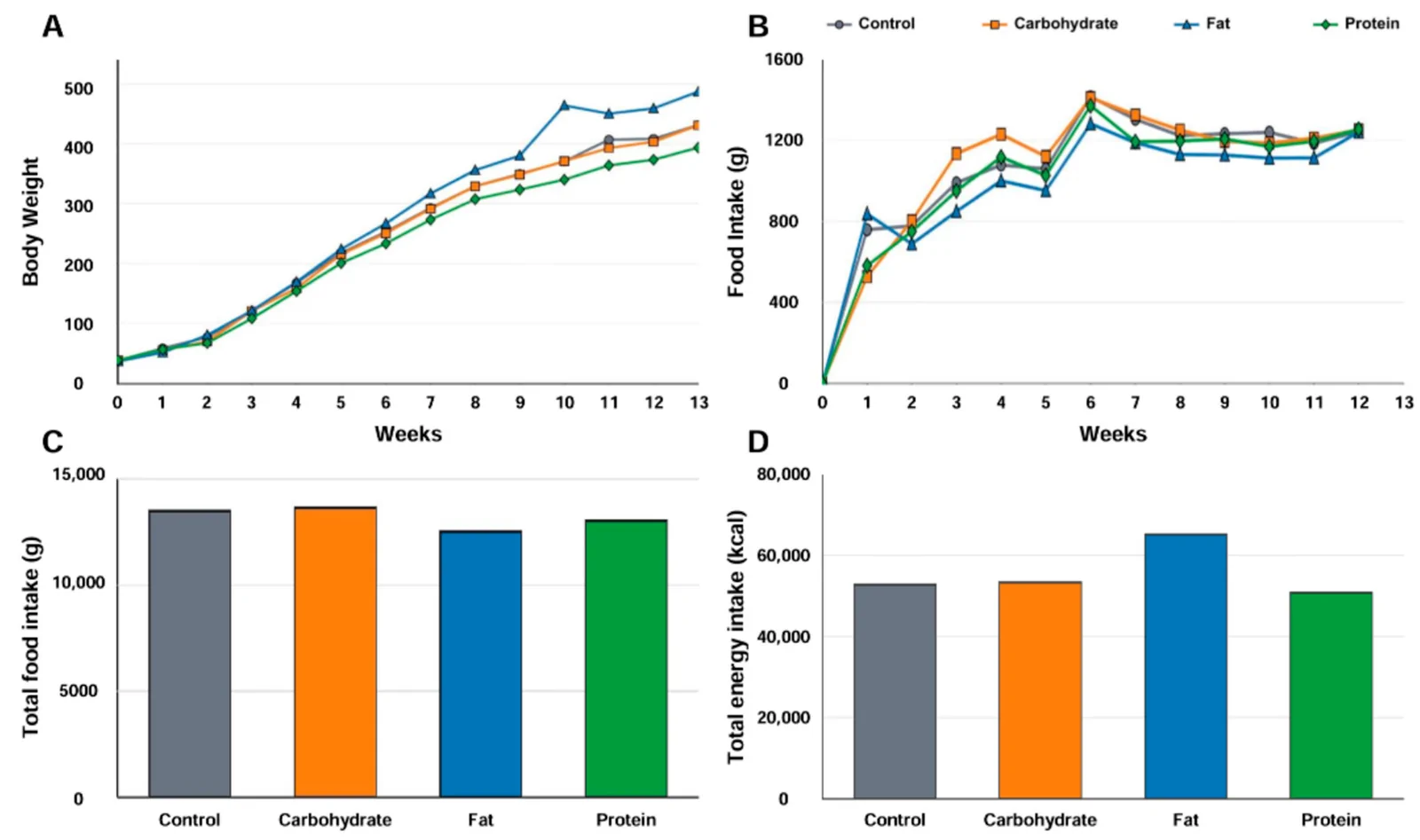
Effects of diets with distinct macronutrient compositions on body weight dynamics, food intake and energy intake in rats over 13 weeks. (**A**) Weekly group mean body weight (g; initially 10 animals per group). The curves provide a descriptive account of growth. (**B**) Weekly food intake (g). (**C**) Cumulative food intake (g). (**D**) Cumulative energy intake (kcal), calculated by multiplying the total food mass in panel C by 3.9 kcal/g for the control, carbohydrate and protein groups and by 5.2 kcal/g for the fat group. Panels (**B**–**D**) show group-level totals.

The effects of diets with distinct macronutrient compositions (control, carbohydrate, fat, and protein) on body weight trajectories, weekly food consumption, and cumulative food intake over the 13-week period are shown in Figure 1. At baseline (Week 0), mean body weights were descriptively similar across groups. All groups gained weight throughout the intervention. The fat-fed group showed the greatest final mean body weight, the carbohydrate and control groups followed similar trajectories, and the protein-fed group showed the smallest gain.

Weekly group-level food intake increased rapidly from Week 0 through Week 6 and then remained relatively stable during Weeks 7–12 (Figure 1B). Cumulative food mass was highest in the carbohydrate group and lowest in the fat group (Figure 1C). Cumulative energy intake, calculated by multiplying the total consumed food mass by dietary energy density, was 52,614.9, 53,149.2, 65,031.2, and 50,684.4 kcal in the control, carbohydrate, fat, and protein groups, respectively (Figure 1D; Supplementary Table S7).

Descriptively, the control and carbohydrate-enriched groups had virtually identical mean body weights at baseline (38.1 and 38.3 g, respectively), at study completion (430.9 and 430.5 g), and for total weight gain (392.8 and 392.2 g). Cumulative group-level food intake was not lower in the carbohydrate-enriched group than in the control group (13,628 and 13,491 g, respectively) (Figure 1).

### 3.2. Markers of Oxidative Stress and Antioxidant Status in Liver Tissue

**Figure 2:**
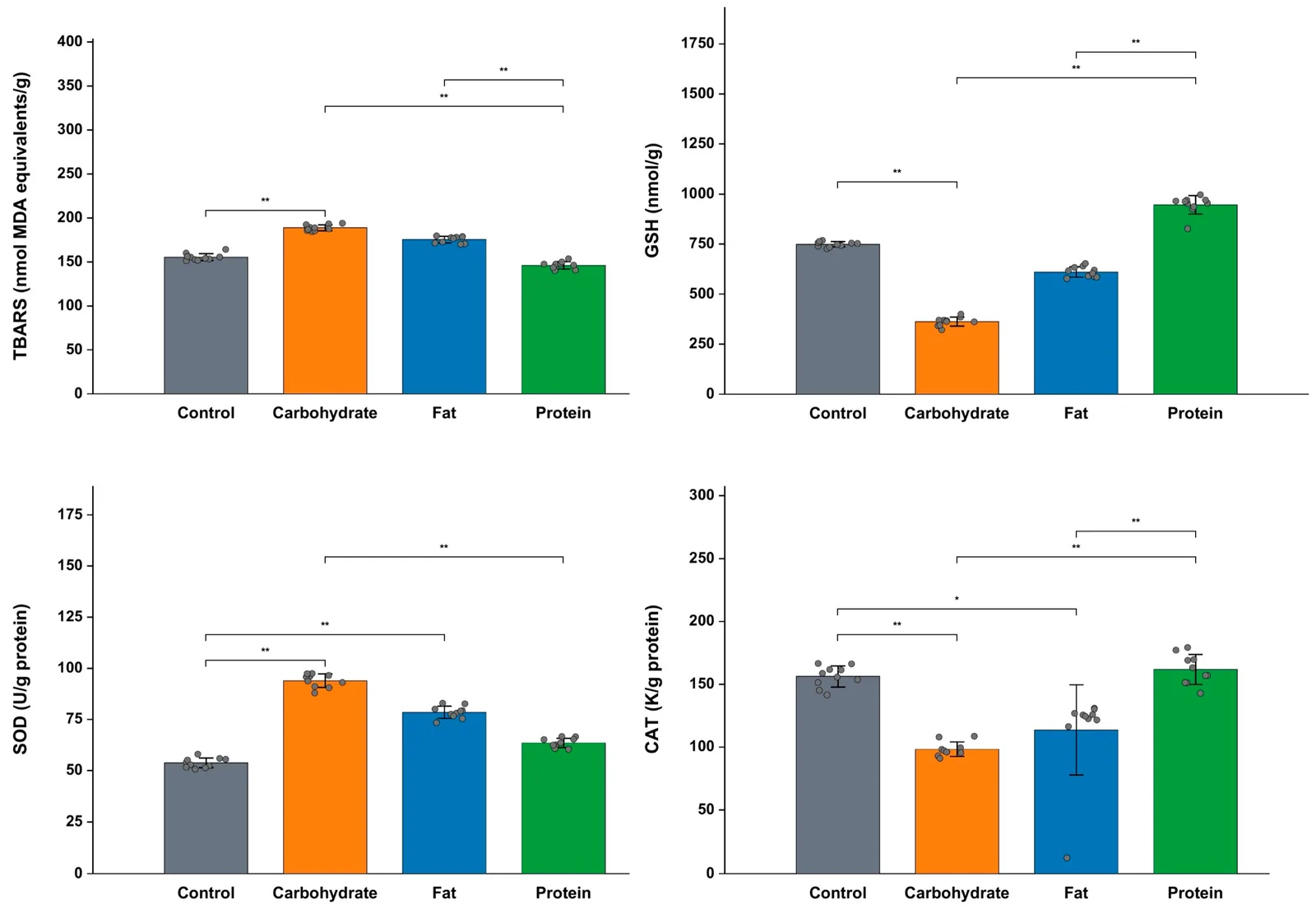
Effects of distinct diets on hepatic oxidative-stress-related parameters. The graphs show liver TBARS, reduced GSH, SOD activity, and CAT activity in control, carbohydrate, fat, and protein groups. Data are presented as mean ± SD with individual data points overlaid (*n* = 10 rats per group). Overall group differences were assessed using the Kruskal–Wallis test, followed by Bonferroni-adjusted Dunn tests for pairwise comparisons. TBARS is expressed as nmol MDA equivalents per gram of liver tissue and GSH as nmol per gram of liver tissue. * *p* < 0.05, ** *p* < 0.01. TBARS: thiobarbituric acid-reactive substances; GSH: reduced glutathione; SOD: superoxide dismutase; CAT: catalase.

Significant overall group differences were detected for liver TBARS, reduced GSH, SOD, and CAT (Figure 2). The carbohydrate group showed the highest TBARS and SOD values, followed by the fat group. Reduced GSH was lower in the carbohydrate group. GSH and CAT values in the protein group were close to or higher than the corresponding control values.

**Figure 3:**
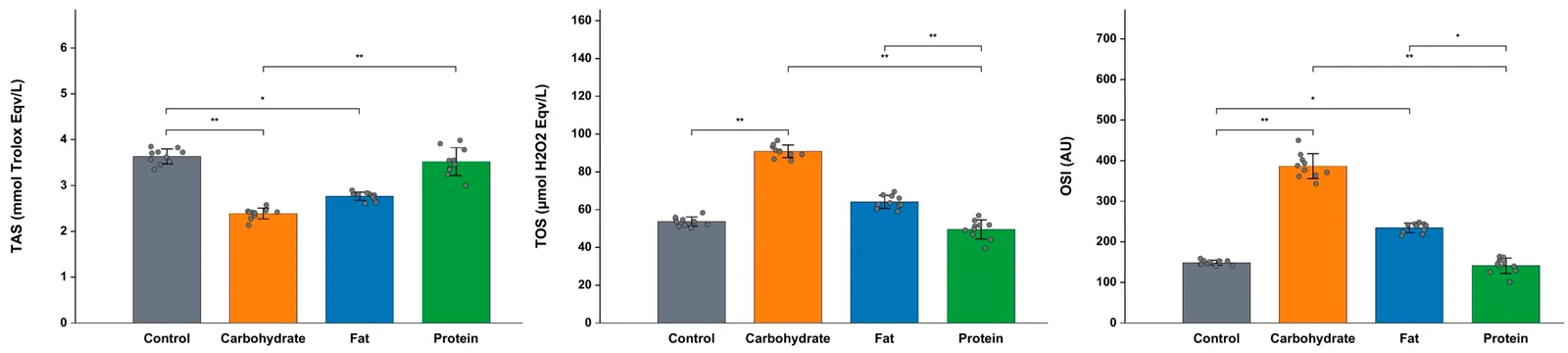
Effects of specific macronutrient diets on liver TAS, TOS and OSI. TAS is expressed as mmol Trolox Eq/L, TOS as μmol H_2_O_2_ Eq/L, and OSI as arbitrary units. Data are presented as mean ± SD with individual data points overlaid (*n* = 10 rats per group). Overall group differences were assessed using the Kruskal–Wallis test, followed by Bonferroni-adjusted Dunn tests for pairwise comparisons. * *p* < 0.05, ** *p* < 0.01. Total antioxidant status: TAS; total oxidant status: TOS; oxidative stress index: OSI.

Significant overall group differences were found for TAS, TOS, and OSI (Figure 3). The carbohydrate group showed the lowest TAS and the highest TOS and OSI values. The fat group showed an intermediate redox profile, whereas protein group values were closer to control values (Figure 3).

### 3.3. Serum Liver Enzyme Levels

**Figure 4:**
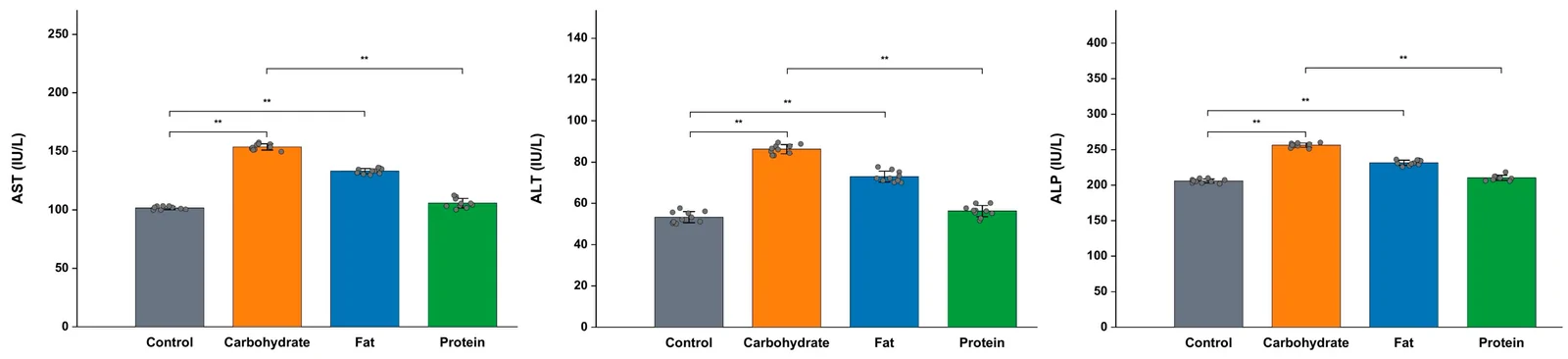
Effect of different macronutrient diets on liver enzyme activities. The graphs show serum activities of AST, ALT, and ALP for control, high-carbohydrate, high-fat, and high-protein diet groups. Data are presented as mean ± SD with individual data points overlaid (*n* = 10 rats per group). Statistical significance was determined using a Kruskal–Wallis analysis to compare the groups because the data were not normally distributed. The Dunn test with Bonferroni correction was used for post hoc comparisons of variables showing differences. ** *p* < 0.01. Aspartate aminotransferase: AST; alanine aminotransferase: ALT; alkaline phosphatase: ALP;.

Serum AST, ALT, and ALP activities differed significantly among groups (Figure 4). The carbohydrate group showed the highest AST, ALT, and ALP levels. Values in the fat group were intermediate, whereas those in the protein group were closer to control values (Figure 4).

### 3.4. Histopathological Assessment of Liver Injury

**Figure 5:**
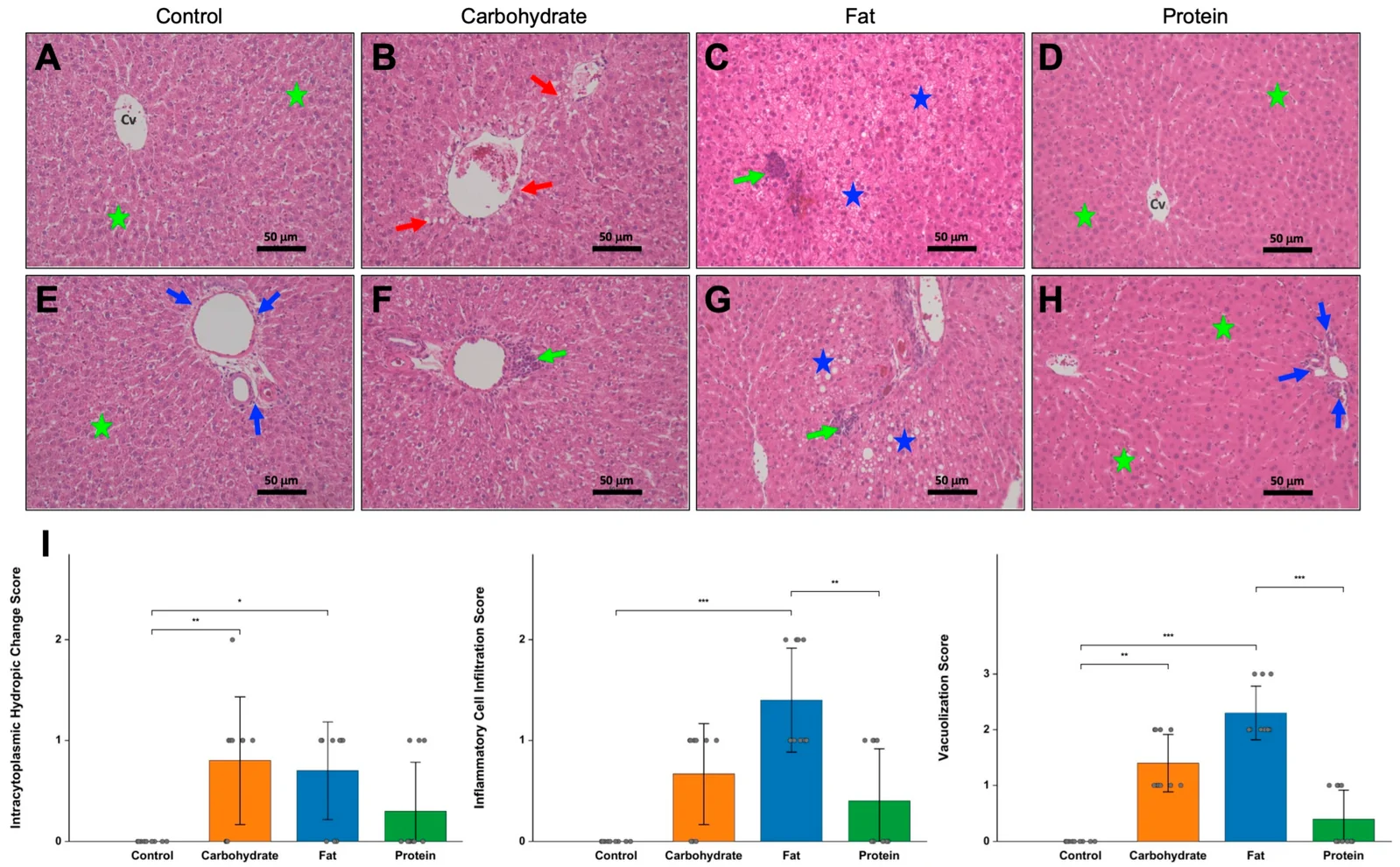
Representative H&E-stained liver sections from the experimental groups (×20). (**A**) The control group shows normal hepatic parenchyma (green asterisks) and a central vein (Cv). (**B**) The carbohydrate-rich diet group shows vacuolization of pericentral hepatocytes (red arrows). (**C**) The fat-rich diet group shows hepatocellular vacuolization (blue asterisks) and inflammatory cell infiltration (green arrow). (**D**) The protein-rich diet group shows normal hepatic parenchyma (green asterisks) and the central vein (Cv). (**E**) The control group shows normal hepatic parenchyma (green asterisk) and the portal area (blue arrows). (**F**) The carbohydrate-rich diet group showing periportal inflammatory cell infiltration (green arrow). (**G**) The fat-rich diet group shows hepatocellular vacuolization (blue asterisks) and inflammatory cell infiltration (green arrow). (**H**) The protein-rich diet group shows normal hepatic parenchyma (green asterisks) and a portal area (blue arrows). (**I**) The graphs show semi-quantitative scoring of intracytoplasmic hydropic change, inflammatory cell infiltration, and vacuolization in liver sections from rats fed control, carbohydrate, fat, and Protein diets. Data are presented as mean ± SD with individual data points overlaid. Statistical significance was determined using the Kruskal–Wallis analysis to compare the groups because the data were not normally distributed. The Dunn test with Bonferroni correction was used for post hoc comparisons of variables showing differences. * *p* < 0.05, ** *p* < 0.01, *** *p* < 0.001.

H&E staining showed preserved hepatic architecture in the control group, without hydropic change, inflammatory cell infiltration, or vacuolization (Figure 5A,E). The carbohydrate group demonstrated pericentral hepatocyte vacuolization and periportal inflammatory cell infiltration (Figure 5B,F). In the fat group, vacuolization was more widespread and was accompanied by inflammatory cell infiltration (Figure 5C,G). The protein group largely retained normal parenchymal organization, with only mild reactive changes (Figure 5D,H). Although limited reactive changes were observed in some areas, these remained quite mild compared with the damage pattern in the fat and carbohydrate groups.

Histological scores differed significantly among groups for intracytoplasmic hydropic change, inflammatory cell infiltration, and vacuolization (Figure 5I). The absence of lesions in the control group indicates preserved liver architecture, while the increase in hydropic changes and vacuolization in the carbohydrate and fat groups supports hepatocyte degeneration. The higher scores for inflammatory cell infiltration and vacuolization in the fat group support a pattern of parenchymal vacuolization and inflammatory morphology associated with the fat-enriched formulation (Figure 5I). The low scores in the protein group support the relative structural preservation of liver tissue. Vacuolization on H&E is not a chemically specific measure of lipid or glycogen content and was interpreted in its morphological context alongside the TEM findings.

### 3.5. Immunohistochemical Findings for IL-6 and TNF-α

**Figure 6:**
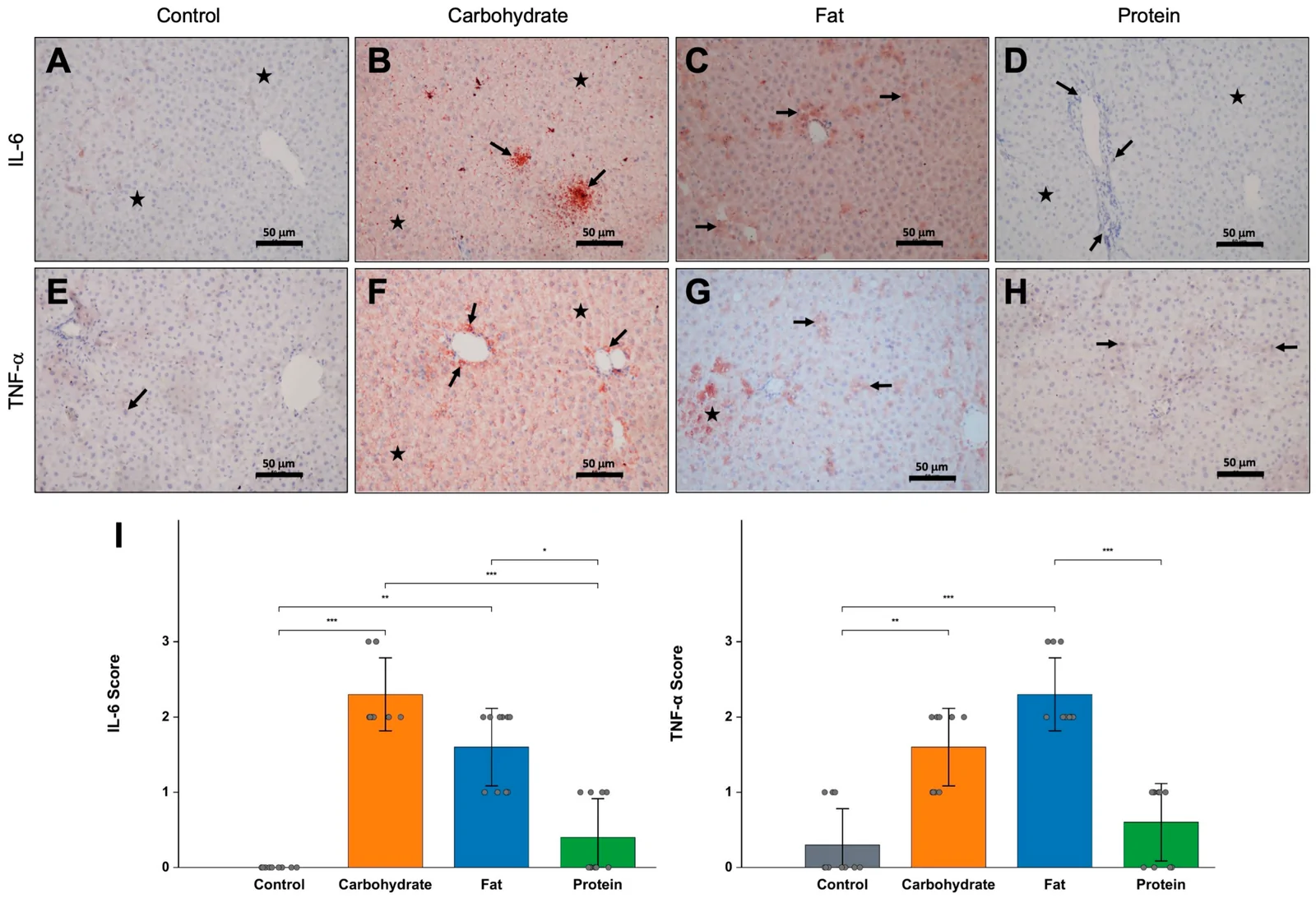
Representative immunohistochemical (IHC) micrographs of IL-6 and TNF-α staining in liver sections from the experimental groups (×20 magnification). (**A**) In the control group, IL-6 immunoreactivity was not prominent **i**n the hepatic parenchyma (asterisks). (**B**) The carbohydrate-rich diet group shows strong to intense focal IL-6 immunoreactivity (arrows), as well as weak immunoreactivity in hepatocytes (asterisks). (**C**) The fat-rich diet group shows moderate IL-6 immunoreactivity in hepatocytes (arrows). (**D**) The protein-rich diet group shows negative IL-6 immunoreactivity in the hepatic parenchyma (asterisks) and the portal area (arrows). (**E**) The control group shows weak TNF-α immunoreactivity in hepatocytes (arrows). (**F**) The carbohydrate-rich diet group shows moderate TNF-α immunoreactivity in the hepatic parenchyma (asterisks) and strong to intense TNF-α immunoreactivity in pericentral hepatocytes (arrows). (**G**) The fat-rich diet group shows moderate TNF-α immunoreactivity in the hepatic parenchyma (arrows) and strong to intense focal TNF-α immunoreactivity in hepatocytes (asterisk). (**H**) The protein-rich diet group shows weak TNF-α immunoreactivity in hepatocytes (arrows). (**I**) The graphs show immunohistochemical scoring of IL-6 and TNF-*α* expression in liver tissues of rats across control, carbohydrate, fat, and protein groups. Data are presented as mean ± SD with individual data points overlaid (*n* = 10 rats per group). Overall group differences were assessed using the Kruskal–Wallis test followed by Bonferroni-adjusted Dunn tests. * *p* < 0.05, ** *p* < 0.01, *** *p* < 0.001. Brown cytokine-associated chromogen was distinguished from the blue haematoxylin nuclear counterstain; the counterstain was not scored as nuclear IL-6 or TNF-α immunoreactivity. Interleukin: IL; tumor necrosis factor alpha: TNF-α.

IL-6 immunoreactivity was not prominent in the control group; low-level TNF-α immunoreactivity was observed (Figure 6A,E). In the carbohydrate group, IL-6 staining showed strong positive immunoreactivity in foci within the liver parenchyma, with weaker staining in the surrounding hepatocytes (Figure 6B). Moderate-intensity IL-6 positive immunoreactivity was observed in hepatocytes in the fat group, whereas IL-6 immunoreactivity was not prominent in the parenchymal or portal areas of the protein group (Figure 6C,D). TNF-α staining in the carbohydrate group showed moderate-intensity immunoreactivity in the parenchyma and strong immunoreactivity in pericentral hepatocytes (Figure 6F). In the fat group, moderate parenchymal and strong focal TNF-α positivity was observed (Figure 6G). Weak but detectable TNF-α immunoreactivity was observed in hepatocytes in the protein group (Figure 6H).

IL-6 and TNF-α scores differed significantly among groups (Figure 6I). IL-6 immunoreactivity was more prominent in the carbohydrate group, whereas TNF-α immunoreactivity was more prominent in the fat group.

### 3.6. Diet-Associated Ultrastructural Alterations in Hepatocytes

**Figure 7:**
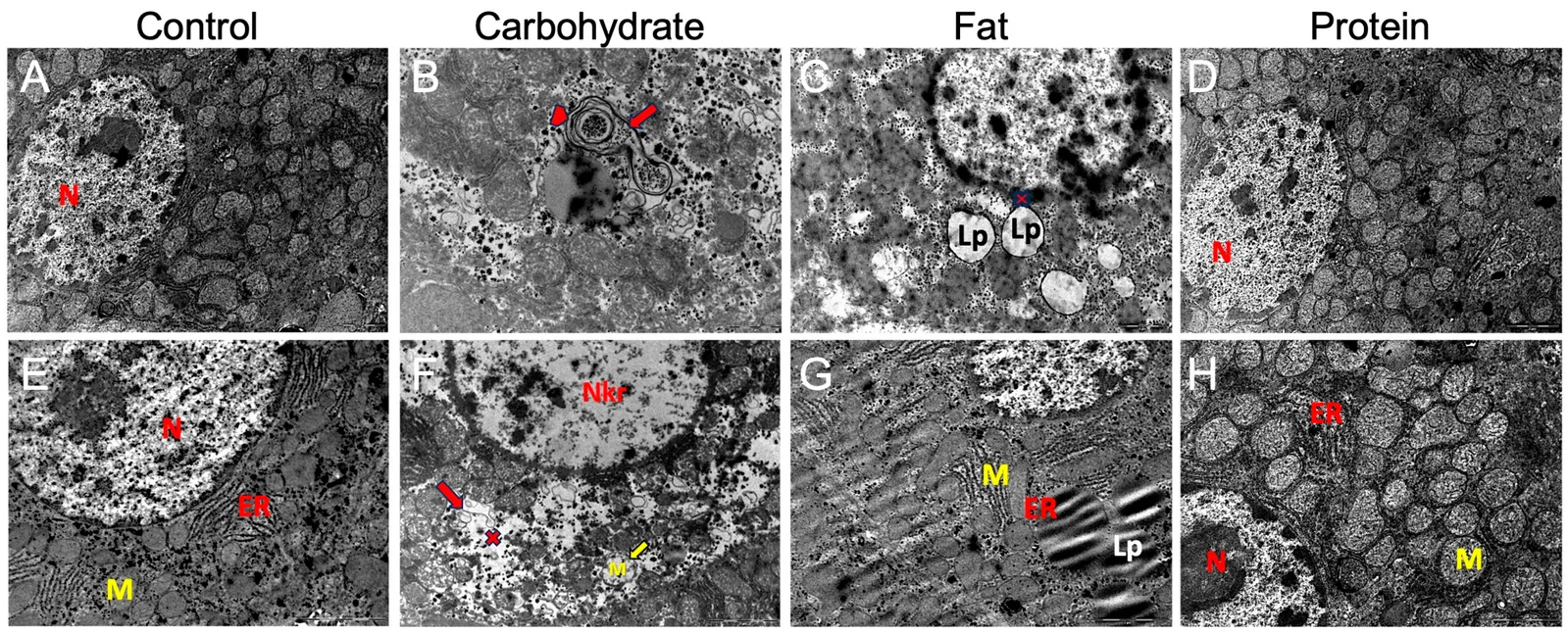
Representative TEM analysis of hepatocytes from the experimental groups. (**A**,**E**) Control group: The hepatocytes exhibited a preserved ultrastructural architecture, including a euchromatic nucleus (N), regularly shaped mitochondria (M), and an organized endoplasmic reticulum (ER). (**A**) ×6300; (**E**) ×10,000. (**B**,**F**) Carbohydrate-rich diet group: Significant ultrastructural changes were observed, including myelin figures (red arrows), endophagosomal structures (arrowheads), chromatin alterations, perinuclear and cytoplasmic edema (×), mitochondrial swelling (M), and partial or complete loss of cristae, as well as intramitochondrial condensation (yellow arrows). (**B**) ×12,500; (**F**) ×6300. (**C**,**G**) Fat-rich diet group: Hepatocytes showed prominent lipid droplet accumulation (Lp), mitochondrial swelling (M), perinuclear and cytoplasmic edema (×), and dilatation and disorganization of the ER. (**C**,**G**) ×6300. (**D**,**H**) Protein-rich diet group: The hepatocytes largely preserved their ultrastructural integrity, with a nucleus (N) that appeared normal, mitochondria (M), and a well-organized ER. (**D**) ×6300; (**H**) ×10,000.

The TEM examination revealed that the ultrastructural organization of hepatocytes in the control group was intact (Figure 7A). The nucleus exhibited euchromatic characteristics, and the mitochondria were observed to have a regular shape and a distinct cristae structure (Figure 7E). The endoplasmic reticulum showed a regular arrangement, and there was no significant edema, swelling of the organelles, or degenerative structure in the cytoplasm (Figure 7E). In the carbohydrate group, however, significant ultrastructural damage was evident (Figure 7B,F). Myelin figures and endolysosomal structures were observed in hepatocytes. In the nucleus, chromatin changes and chromatolysis were evident, and perinuclear and cytoplasmic edema became prominent. Swelling of the mitochondria, loss of cristae, and intramitochondrial condensation indicated structural alterations at the organelle level (Figure 7B,F).

In the fat group, prominent lipid droplet accumulation was observed in hepatocytes. These lipid droplets formed the ultrastructural counterpart of the vacuolization observed in H&E sections (Figure 7C,G). Mitochondrial swelling and endoplasmic reticulum dilation/disorganization were part of the ultrastructural pattern observed alongside lipid droplets (Figure 7C,G). In contrast, TEM analysis of the protein group revealed that the ultrastructure of hepatocytes was largely preserved (Figure 7D,H). The nucleus appeared normal, the mitochondria were arranged regularly, and the endoplasmic reticulum was organized (Figure 7D,H). No significant accumulation of lipid droplets, swelling of the mitochondria, loss of cristae, or widespread cytoplasmic edema was observed.

## 4. Discussion

In this study, dietary formulations administered during post-weaning growth were associated with distinct hepatic biochemical and morphological patterns. Redox changes and mitochondrial–nuclear ultrastructural findings predominated in the carbohydrate group, whereas vacuolization, lipid droplet accumulation, and inflammatory findings predominated in the fat group. Hepatocyte structure was largely preserved in the protein group, and many biochemical values remained close to control values. The controlled design, involving the same rat strain, sex, age at diet initiation, housing conditions, and 13-week exposure, enabled biochemical, histological, immunohistochemical, and ultrastructural comparisons of hepatic responses associated with the three dietary patterns within the same experiment.

Dietary energy density should be considered together with the consumed food mass. The cumulative energy calculation showed that the fat group had the highest energy intake despite consuming less food mass. The hepatic findings in this group are therefore interpreted in relation to the complete fat-enriched formulation and energy intake under ad libitum feeding. The developmental stage is also relevant: post-weaning rats have shown greater susceptibility than young-adult rats to high-fat-diet-associated metabolic and hepatic changes [36]. All groups comprised male Wistar albino rats of the same starting age and were maintained under the same housing conditions. This shared experimental framework supports comparison of diet-associated hepatic phenotypes during ongoing somatic growth.

The redox pattern in the carbohydrate group requires joint consideration of lipid peroxidation and antioxidant defense. Increased TBARS is consistent with an increase in thiobarbituric acid-reactive lipid peroxidation products, whereas lower GSH and TAS are consistent with changes in antioxidant reserves. SOD and CAT activities reflect the antioxidant enzyme response in this setting. The co-occurrence of this biochemical pattern with mitochondrial and nuclear changes provides a redox context for the structural findings in the hepatic parenchyma. High-sucrose and fructose models have likewise been associated with increased liver enzymes, lipid vacuoles, and mitochondrial alterations [37,38]; altered hepatic metabolism and structural integrity have been reported in high-starch models [39]. Ferramosca et al. showed that high-carbohydrate and high-fat diets affect hepatic lipogenesis differently [10]. The effects of starch and fiber content on hepatic lipid homeostasis in purified diets also support the importance of dietary composition [19]. Joint consideration of the starch-derived substrate load and the accompanying protein change is therefore relevant to explaining the observed response under the present formulation.

The control and carbohydrate formulations have similar compositions; the principal change is an increase in corn starch accompanied by a decrease in casein. The control-to-carbohydrate contrast should be interpreted as the effect of the complete isocaloric carbohydrate-for-protein substitution rather than as an isolated carbohydrate main effect. This substitution changes the carbohydrate-derived substrate load together with protein and amino acid supply, requiring the redox response to be considered within this nutritional balance. The 18 kcal% protein level was lower than that of the control, but it remained well above levels commonly used to induce experimental protein restriction. For context, AIN-93G contains 200 g casein and 3 g L-cystine per kg for growth, whereas the present carbohydrate formulation normalizes to approximately 175 g casein and 2.9 g L-cystine per kg [40,41,42]. AIN-93G is used here as a nutritional reference point and not as a claim that the present formulation was AIN-93G-compliant. Redox changes predominated in the carbohydrate group, whereas vacuolization and lipid droplet accumulation predominated in the fat group, identifying distinct biochemical and morphological patterns associated with the dietary formulations. Given that the fat formulation derived 20% of its energy from protein and was therefore not an isonitrogenous comparator, these findings support formulation-specific hepatic responses and the interpretation of the observed phenotype at the level of the complete formulation, including the carbohydrate-for-protein substitution.

The fat group showed a different predominant pattern. Vacuolization, inflammatory cell infiltration, TEM-observed lipid droplets, and TNF-*α* immunoreactivity were prominent, consistent with reports from high-fat-diet models [43,44,45]. Increased liver volume and lipid accumulation have also been reported with high-fat diets [18]. Such changes in tissue composition may affect the magnitude of measurements normalized per gram of tissue; measurements normalized to protein additionally require consideration of protein content. A possible lipid dilution effect is an explanation that may be considered within this framework for the biochemical pattern in the fat group. Most protein group outcomes remained closer to control values. In this formulation, increased casein was accompanied by reduced carbohydrate, changing amino acid supply and carbohydrate-derived metabolic load together. Casein-derived amino acids may contribute to protein turnover and the substrate pool for glutathione synthesis, while the simultaneous reduction in carbohydrate may alter lipogenic substrate flux. Lacroix et al. reported modest increases in glutathione and glutathione-S-transferase levels and lower adipose tissue accumulation during long-term high-protein feeding [12]. The differing findings of previous high-protein-diet studies indicate the combined importance of protein amount and source, energy balance, duration, and metabolic background [46,47,48].

The immunohistochemical and ultrastructural findings provide tissue-level context for the biochemical results. The distribution of IL-6 and TNF-α immunoreactivity, together with vacuolization and cellular infiltration on H&E, defines formulation-associated inflammatory patterns. The preserved TEM appearance in the protein group is also consistent with the preserved parenchymal structure on H&E and low histological and IHC scores. IL-6 and TNF-*α* were assessed using semi-quantitative immunoreactivity scores, and TEM documented organelle morphology. Functional assessment of energy production, protein processing, and specific signaling pathways is a subject for mechanistic studies that complement these structural findings. Serum AST, ALT, and ALP activities were considered supportive biochemical indicators alongside the histological and ultrastructural findings. Extrahepatic sources of AST and ALP and the potential contribution of bone-derived ALP during growth were considered in this interpretation [49]. Serum LDH and GLDH address complementary cell-injury questions; GLDH has been investigated as a more liver-specific biomarker where transaminases may have extrahepatic contributions [50].

The principal contribution of this study is comparative and integrative. The early-life framing also defines the appropriate translational scope. Nutritional exposures during immediate postnatal life can program later metabolic phenotypes in experimental animals, and this concept has potential relevance to human growth and long-term metabolic health [2]. Nevertheless, rat and human developmental timelines are not linearly interchangeable, and age, body weight, and developmental stage must be considered explicitly when translating rodent findings [20]. The findings therefore experimentally characterize hepatic phenotypes accompanying dietary exposures initiated after weaning and provide a comparative framework for developmental nutrition research.

The scope and limitations of the study should be considered when interpreting the findings. Only male rats were included, requiring the results to be considered within this sex; sex hormones can modify susceptibility to diet-induced liver injury in rodents [51]. Inflammation was assessed by semi-quantitative IL-6 and TNF-*α* immunohistochemistry, and organelle changes were assessed by TEM morphology. The potential contribution of residual blood to SOD and CAT activities following preparation of liver tissue by saline rinsing should also be considered. Group-level food-intake and body weight records enabled descriptive assessment of energy and growth patterns. The absence of whole-liver mass/volume and quantitative hepatic lipid assessment limits direct evaluation of diet-associated organ enlargement and a possible lipid dilution effect. Studies combining organ weight ratios, hepatic triglyceride/glycogen quantities, and specific metabolic pathways may advance the mechanistic explanation of these morphological and biochemical patterns. Joint planning of histological and functional outcomes would extend the scope of these comparisons in accordance with experimental reporting principles [52]; assessment of lipid metabolism regulators such as PPARα/PPARγ may also provide complementary information [53].

The control and carbohydrate-enriched diets were formulated as a reciprocal isocaloric exchange in which the six-percentage-point increase in carbohydrate necessarily accompanied a six-percentage-point decrease in protein. Consequently, the present design estimates the hepatic phenotype of the complete 70 kcal% carbohydrate formulation and cannot fully partition the contribution of increased carbohydrate from that of reduced protein. A future factorial design that varies carbohydrate and protein independently while controlling energy density, protein quality, essential amino acid supply, and individual energy intake would be required for nutrient-specific causal separation. This approach would allow the nutrient-specific hepatic phenotypes identified in the present comparison to be distinguished.

## 5. Conclusions

Under the conditions of this 13-week study in male Wistar rats, the carbohydrate-enriched diet was associated with the largest unfavorable shifts in hepatic redox measures, serum liver enzymes, and ultrastructural morphology. The fat-enriched diet showed more prominent vacuolization, TEM-observed lipid droplets, and inflammatory findings, whereas many findings in the protein group were closer to control values. The study provides a controlled, multilayered comparison of diet-associated hepatic phenotypes during post-weaning growth but does not establish nutrient-specific molecular mechanisms or directly measure liver growth. Translation to human growth should therefore remain developmental and hypothesis-generating rather than age-equivalent or clinical.

## Data Availability

Data supporting the reported findings are contained within the article and Supplementary Materials. Additional raw data and original statistical output are available from the corresponding author on reasonable request.

## Supplementary Materials

The following supporting information can be downloaded at: https://www.mdpi.com/article/10.3390/metabo16100781/s1, Supplementary Table S1 presents dietary composition; Supplementary Tables S2–S5 report biochemical, histological and immunohistochemical results. Supplementary Table S6 and Supplementary Figure S1 show weekly group-mean body weights, and Supplementary Table S7 presents food intake and calculated energy intake.

## Abbreviations

The following abbreviations are used in this manuscript:

ALP	Alkaline phosphatase
ALT	Alanine aminotransferase
AST	Aspartate Aminotransferase
CAT	Catalase
GSH	Glutathione (Reduced)
H&E	Hematoxylin and Eosin
IL-6	Interleukin 6
OSI	Oxidative stress index
SOD	Superoxide dismutase
TAS	Total antioxidant status
TBARS	Thiobarbituric acid-reactive substances
TEM	Transmission electron microscopy
TOS	Total oxidant status
TNF-α	Tumor Necrosis Factor-alpha
