# Supplementary material for: Dietary Macronutrient Composition Is Associated with Distinct Hepatic Phenotypes During Post-Weaning Growth: An Integrated Biochemical, Histological, Immunohistochemical and Ultrastructural Study
Source: Metabolites. 2026 Oct 15;16(10):781. doi: 10.3390/metabo16100781 (PMC13707318; doi:10.3390/metabo16100781)
Supplement: Supplementary file 1 [file metabolites-16-00781-s001.zip › metabolites-4585470-supplementary.pdf]

**Supplementary Table S1.** Composition, formulation and energy values of the experimental diets

| Diet | Control | | 70 kcal% Carbohydrate | | 60 kcal% Fat | | 50 kcal% Protein | |
|---|---|---|---|---|---|---|---|---|
| | g% | kcal% | g% | kcal% | g% | kcal% | g% | kcal% |
| **Protein** | 24 | 24 | 18 | 18 | 26 | 20 | 49 | 50 |
| **Carbohydrate** | 62 | 64 | 68 | 70 | 26 | 20 | 37 | 38 |
| **Fat** | 5 | 12 | 5 | 12 | 35 | 60 | 5 | 12 |
| **Total** | 91 | 100 | 91 | 100 | 87 | 100 | 91 | 100 |
| **Energy density (kcal/g)** | 3.9 | | 3.9 | | 5.2 | | 3.9 | |
| **Ingredient** | **Control** | | **70 kcal% Carbohydrate** | | **60 kcal% Fat** | | **50 kcal% Protein** | |
| | **g (g%)** | **kcal (kcal%)** | **g (g%)** | **kcal (kcal%)** | **g (g%)** | **kcal (kcal%)** | **g (g%)** | **kcal (kcal%)** |
| Casein | 243.3 (23.3) | 973 (24.0) | 182.5 (17.5) | 730 (18.0) | 200 (25.8) | 800 (19.7) | 507 (48.5) | 2028 (50.0) |
| L-Cystine | 3 (0.3) | 12 (0.3) | 3 (0.3) | 12 (0.3) | 3 (0.4) | 12 (0.3) | 3 (0.3) | 12 (0.3) |
| Corn starch | 445 (42.6) | 1780 (43.9) | 505.6 (48.4) | 2022 (49.8) | – | – | 181.3 (17.3) | 725 (17.9) |
| Maltodekstrin 10 | 125 (12.0) | 500 (12.3) | 125 (12.0) | 500 (12.3) | 125 (16.2) | 500 (12.3) | 125 (12.0) | 500 (12.3) |
| Sucrose | 68.8 (6.6) | 275 (6.8) | 68.8 (6.6) | 275 (6.8) | 68.8 (8.9) | 275 (6.8) | 68.8 (6.6) | 275 (6.8) |
| Cellulose, BW200 | 50 (4.8) | – | 50 (4.8) | – | 50 (6.5) | – | 50 (4.8) | – |
| Soybean oil | 25 (2.4) | 225 (5.5) | 25 (2.4) | 225 (5.5) | 25 (3.2) | 225 (5.5) | 25 (2.4) | 225 (5.5) |
| Lard | 28 (2.7) | 252 (6.2) | 28 (2.7) | 252 (6.2) | 245 (31.7) | 2205 (54.4) | 28 (2.7) | 252 (6.2) |
| Mineral mix S10026 | 10 (1.0) | – | 10 (1.0) | – | 10 (1.3) | – | 10 (1.0) | – |
| Dicalcium phosphate | 13 (1.2) | – | 13 (1.2) | – | 13 (1.7) | – | 13 (1.2) | – |
| Calcium carbonate | 5.5 (0.5) | – | 5.5 (0.5) | – | 5.5 (0.7) | – | 5.5 (0.5) | – |
| Potassium citrate, monohydrate | 16.5 (1.6) | – | 16.5 (1.6) | – | 16.5 (2.1) | – | 16.5 (1.6) | – |
| Vitamin mix, V10001 | 10 (1.0) | 40 (1.0) | 10 (1.0) | 40 (1.0) | 10 (1.3) | 40 (1.0) | 10 (1.0) | 40 (1.0) |
| Choline bitartrate | 2 (0.2) | – | 2 (0.2) | – | 2 (0.3) | – | 2 (0.2) | – |
| FD&C Yellow Dye No. 5 | 0.025 (0.0) | – | 0.05 (0.0) | – | – | – | – | – |
| FD&C Red Dye No. 40 | 0.025 (0.0) | – | – | – | – | – | 0.05 (0.0) | – |
| FD&C Blue Dye No. 1 | – | – | – | – | 0.05 (0.0) | – | – | – |
| **Reported total** | **1045.2 (100.0)** | **4057 (100.0)** | **1045.0 (100.0)** | **4057 (100.0)** | **773.85 (100.0)** | **4057 (100.0)** | **1045.2 (100.0)** | **4057 (100.0)** |

g%: percentage of total diet mass; kcal%: percentage of total energy. Upper g% totals cover the three macronutrients. Lower percentages use the reported column totals in the final row. "–" denotes zero quantity or energy contribution. Ingredient quantities and totals follow source [23]; small differences between ingredient sums and reported totals reflect rounding.

**Supplementary Table S2.** Effects of distinct diets on hepatic oxidative-stress-related parameters (the same data are presented in Figure 2).

| Variable | Group | Mean ± SD | M(Q1–Q3) | (Min–Max) | Kruskal–Wallis H | Source p | Effect size η²_H |
|---|---|---|---|---|---|---|---|
| TBARS (nmol MDA equivalents/g) | Control [ab] | 155.5 ± 4.0 | 154.8(152.4-157.2) | 151.5 - 164.2 | *36.023* | *0.001** | 0.917 |
| | Carbohydrate [c] | 188.8 ± 3.4 | 188.2(185.6-192.6) | 184.5 - 194.0 | | | |
| | Fat [ac] | 175.4 ± 3.8 | 177.2(171.2-178.6) | 170.0 - 179.6 | | | |
| | Protein [b] | 146.0 ± 4.2 | 146.9(142.4-148.5) | 140.1 - 153.6 | | | |
| GSH (nmol/g) | Control [ab] | 749.0 ± 12.9 | 750.8(738.8-758.3) | 726.7 - 768.3 | *36.592* | *0.001** | 0.933 |
| | Carbohydrate [c] | 362.5 ± 22.2 | 365.0(344.2-373.8) | 321.7 - 399.8 | | | |
| | Fat [ac] | 610.8 ± 25.9 | 610.2(587.5-635.0) | 578.0 - 653.3 | | | |
| | Protein [b] | 946.2 ± 46.3 | 959.2(933.8-970.0) | 826.7 - 996.7 | | | |
| SOD (U/g protein) | Control [a] | 53.7 ± 2.4 | 53.4(51.5-55.6) | 50.7 - 58.0 | *36.585* | *0.001** | 0.933 |
| | Carbohydrate [b] | 93.9 ± 3.3 | 94.7(90.9-96.7) | 87.9 - 97.5 | | | |
| | Fat [bc] | 78.5 ± 3.0 | 78.6(76.3-80.7) | 73.4 - 82.9 | | | |
| | Protein [ac] | 63.5 ± 2.3 | 63.1(61.4-65.6) | 60.4 - 66.6 | | | |
| CAT (K/g protein) | Control [a] | 156.2 ± 8.4 | 157.3(149.9-162.8) | 141.5 - 166.6 | *31.857* | *0.001** | 0.802 |
| | Carbohydrate [b] | 98.3 ± 5.8 | 96.7(94.8-101.6) | 91.1 - 108.7 | | | |
| | Fat [b] | 113.7 ± 35.9 | 125.0(120.3-127.7) | 12.4 - 131.0 | | | |
| | Protein [a] | 161.8 ± 12.0 | 160.1(151.3-171.6) | 142.9 - 179.3 | | | |

M denotes the median; Q1 and Q3 denote the first and third quartiles. Overall group differences were assessed using the Kruskal–Wallis test, followed, where applicable, by Bonferroni-adjusted Dunn post hoc comparisons. Groups sharing a letter do not differ significantly; groups without a shared letter differ at Bonferroni-adjusted $p < 0.05$. SD denotes standard deviation. GSH: reduced glutathione; SOD: superoxide dismutase; CAT: catalase; MDA: malondialdehyde. Min: minimum; Max: maximum; H: Kruskal–Wallis test statistic; η²_H: eta-squared effect size based on the Kruskal–Wallis test.

**Supplementary Table S3.** Effects of specific macronutrient diets on liver TAS, TOS and OSI. TAS is expressed as mmol Trolox Eq/L, TOS as μmol $H_2O_2$ Eq/L, and OSI as arbitrary units (the same data are presented in Figure 3).

| Variable | Group | Mean ± SD | M(Q1–Q3) | (Min–Max) | Kruskal–Wallis H | Source p | Effect size η²_H |
|---|---|---|---|---|---|---|---|
| **TAS (mmol Trolox Eqv/L)** | Control[a] | 3.6 ± 0.2 | 3.7(3.5-3.8) | 3.4 - 3.9 | *33.174* | *0.001** | 0.838 |
| | Carbohydrate[b] | 2.4 ± 0.1 | 2.4(2.3-2.4) | 2.1 - 2.6 | | | |
| | Fat [bc] | 2.8 ± 0.1 | 2.8(2.7-2.8) | 2.6 - 2.9 | | | |
| | Protein [ac] | 3.5 ± 0.3 | 3.5(3.3-3.8) | 3.0 - 4.0 | | | |
| **TOS (μmol H2O2 Eqv/L)** | Control [ab] | 53.7 ± 2.4 | 53.7(51.6-55.4) | 50.4 - 58.4 | *33.994* | *0.001** | 0.861 |
| | Carbohydrate [c] | 90.9 ± 3.3 | 90.8(88.6-93.5) | 85.9 - 96.7 | | | |
| | Fat [ac] | 64.1 ± 3.4 | 63.2(61.7-67.4) | 59.1 - 69.5 | | | |
| | Protein [b] | 49.5 ± 5.1 | 50.5(46.2-52.5) | 39.5 - 56.9 | | | |
| **OSI (AU)** | Control[a] | 148.6 ± 6.2 | 149.0(143.3-153.0) | 140.0 - 159.0 | *33.098* | *0.001** | 0.836 |
| | Carbohydrate[b] | 386.5 ± 30.8 | 382.5(363.3-405.3) | 343.0 - 450.0 | | | |
| | Fat [b] | 234.5 ± 11.8 | 240.0(222.5-244.0) | 215.0 - 248.0 | | | |
| | Protein[a] | 141.1 ± 19.0 | 143.5(128.0-156.8) | 101.0 - 164.0 | | | |

M denotes the median; Q1 and Q3 denote the first and third quartiles. Overall group differences were assessed using the Kruskal–Wallis test, followed, where applicable, by Bonferroni-adjusted Dunn post hoc comparisons. Groups sharing a letter do not differ significantly; groups without a shared letter differ at Bonferroni-adjusted $p < 0.05$. SD denotes standard deviation. TOS: total oxidant status; OSI: oxidative stress index. Min: minimum; Max: maximum; H: Kruskal–Wallis test statistic; η²_H: eta-squared effect size based on the Kruskal–Wallis test.

**Supplementary Table S4.** Effect of different macronutrient diets on liver enzyme activities (the same data are presented in Figure 4).

| Variable | Group | Mean ± SD | M(Q1–Q3) | (Min–Max) | Kruskal–Wallis H | Source p | Effect size $\eta^2$_H |
|---|---|---|---|---|---|---|---|
| **AST (IU/L)** | Control[a] | 101.6 ± 1.4 | 101.6(100.4-103.2) | 99.9 - 103.4 | *34.675* | *0.001** | 0.880 |
| | Carbohydrate[b] | 153.7 ± 2.6 | 153.5(151.4-156.2) | 149.7 - 157.7 | | | |
| | Fat[bc] | 133.1 ± 2.3 | 133.3(131.0-135.2) | 129.8 - 136.2 | | | |
| | Protein[ac] | 105.7 ± 4.1 | 104.9(102.8-109.9) | 100.2 - 112.4 | | | |
| **ALT (IU/L)** | Control[a] | 53.3 ± 2.7 | 52.8(51.0-55.8) | 50.2 - 57.7 | *34.180* | *0.001** | 0.866 |
| | Carbohydrate[b] | 86.2 ± 2.2 | 86.4(84.1-88.0) | 83.2 - 89.5 | | | |
| | Fat[bc] | 73.0 ± 2.7 | 72.2(70.9-75.5) | 70.1 - 77.7 | | | |
| | Protein[ac] | 56.3 ± 2.7 | 56.5(54.6-58.3) | 51.7 - 60.2 | | | |
| **ALP (IU/L)** | Control[a] | 206.0 ± 2.8 | 205.8(203.3-208.3) | 202.1 - 210.1 | *34.685* | *0.001** | 0.880 |
| | Carbohydrate[b] | 256.4 ± 3.0 | 256.7(254.7-259.0) | 251.1 - 260.1 | | | |
| | Fat[bc] | 231.6 ± 3.6 | 231.1(228.8-235.3) | 225.8 - 236.3 | | | |
| | Protein[ac] | 210.4 ± 3.7 | 210.0(207.4-212.4) | 205.9 - 218.5 | | | |

M denotes the median; Q1 and Q3 denote the first and third quartiles. Overall group differences were assessed using the Kruskal–Wallis test, followed, where applicable, by Bonferroni-adjusted Dunn post hoc comparisons. Groups sharing a letter do not differ significantly; groups without a shared letter differ at Bonferroni-adjusted $p < 0.05$. SD denotes standard deviation. AST: aspartate aminotransferase; ALT: alanine aminotransferase; ALP: alkaline phosphatase. Min: minimum; Max: maximum; H: Kruskal–Wallis test statistic; $\eta^2$_H: eta-squared effect size based on the Kruskal–Wallis test.

**Supplementary Table S5.** Histopathological and immunohistochemical scores (the same data are presented in Figure 5 and Figure 6).

| Variable | Group | Mean ± SD | M(Q1–Q3) | (Min–Max) | Kruskal–Wallis H | P | Effect size η²_H |
|---|---|---|---|---|---|---|---|
| **Hydropic change** | Control[a] | 0.0 ± 0.0 | 0.0(0.0–0.0) | 0.0 - 0.0 | 13.901 | 0.003* | 0.303 |
| | Carbohydrate[b] | 0.8 ± 0.6 | 1.0(0.3–1.0) | 0.0 - 2.0 | | | |
| | Fat[b] | 0.7 ± 0.5 | 1.0(0.3–1.0) | 0.0 - 1.0 | | | |
| | Protein [ab] | 0.3 ± 0.5 | 0.0(0.0–0.8) | 0.0 - 1.0 | | | |
| **Inflammatory-cell infiltration** | Control[a] | 0.0 ± 0.0 | 0.0(0.0–0.0) | 0.0 - 0.0 | 23.012 | <0.001* | 0.572 |
| | Carbohydrate [ab] | 0.7 ± 0.5 | 1.0(0.0–1.0) | 0.0 - 1.0 | | | |
| | Fat [b] | 1.4 ± 0.5 | 1.0(1.0–2.0) | 1.0 - 2.0 | | | |
| | Protein [a] | 0.4 ± 0.5 | 0.0(0.0–1.0) | 0.0 - 1.0 | | | |
| **Vacuolization** | Control[a] | 0.0 ± 0.0 | 0.0(0.0–0.0) | 0.0 - 0.0 | 32.713 | <0.001* | 0.825 |
| | Carbohydrate [bc] | 1.4 ± 0.5 | 1.0(1.0–2.0) | 1.0 - 2.0 | | | |
| | Fat [b] | 2.3 ± 0.5 | 2.0(2.0–2.8) | 2.0 - 3.0 | | | |
| | Protein [ac] | 0.4 ± 0.5 | 0.0(0.0–1.0) | 0.0 - 1.0 | | | |
| **IL-6 immunoreactivity** | Control[a] | 0.0 ± 0.0 | 0.0(0.0–0.0) | 0.0 - 0.0 | 32.904 | <0.001* | 0.831 |
| | Carbohydrate[b] | 2.3 ± 0.5 | 2.0(2.0–2.8) | 2.0 - 3.0 | | | |
| | Fat [b] | 1.6 ± 0.5 | 2.0(1.0–2.0) | 1.0 - 2.0 | | | |
| | Protein [a] | 0.4 ± 0.5 | 0.0(0.0–1.0) | 0.0 - 1.0 | | | |
| **TNF-α immunoreactivity** | Control[a] | 0.3 ± 0.5 | 0.0(0.0–0.8) | 0.0 - 1.0 | 29.455 | <0.001* | 0.735 |
| | Carbohydrate [bc] | 1.6 ± 0.5 | 2.0(1.0–2.0) | 1.0 - 2.0 | | | |
| | Fat [b] | 2.3 ± 0.5 | 2.0(2.0–2.8) | 2.0 - 3.0 | | | |
| | Protein [ac] | 0.6 ± 0.5 | 1.0(0.0–1.0) | 0.0 - 1.0 | | | |

M denotes the median; Q1 and Q3 denote the first and third quartiles. Overall group differences were assessed using the Kruskal–Wallis test, followed, where applicable, by Bonferroni-adjusted Dunn post hoc comparisons. Groups sharing a letter do not differ significantly; groups without a shared letter differ at Bonferroni-adjusted $p < 0.05$. SD denotes standard deviation. H&E: hematoxylin and eosin; IHC: immunohistochemistry; IL-6: interleukin 6; TNF-α: tumor necrosis factor alpha. Min: minimum; Max: maximum; H: Kruskal–Wallis test statistic; η²_H: eta-squared effect size based on the Kruskal–Wallis test.

**Supplementary Table S6.** Group-mean body weights from baseline to Week 13 (g; the same data are presented in Figure 1A).

| Week | Control | Carbohydrate | Fat | Protein |
|---|---|---|---|---|
| **0** | 38.1 | 38.3 | 36.6 | 38.7 |
| **1** | 58.4 | 55.2 | 51.3 | 56.4 |
| **2** | 76.9 | 69.8 | 80.6 | 67.2 |
| **3** | 120.9 | 120.7 | 121.2 | 108.3 |
| **4** | 167.7 | 158.5 | 169.1 | 153.8 |
| **5** | 217.4 | 215.1 | 223.8 | 200.6 |
| **6** | 252.9 | 250.5 | 266.7 | 233.3 |
| **7** | 292.5 | 291.2 | 316.6 | 273.0 |
| **8** | 328.9 | 328.8 | 355.7 | 307.2 |
| **9** | 349.4 | 348.2 | 379.8 | 323.2 |
| **10** | 370.2 | 370.9 | 463.7 | 339.8 |
| **11** | 406.5 | 392.6 | 449.9 | 363.6 |
| **12** | 408.0 | 403.0 | 458.7 | 373.1 |
| **13** | 430.9 | 430.5 | 486.6 | 393.3 |

**Supplementary Table S7.** Recorded cage-level feed mass and nominal dietary energy.

| Group | Cage 1 (g) | Cage 2 (g) | Group total (g) | kcal/g | Nominal total (kcal) |
|---|---|---|---|---|---|
| **Control** | 6804 | 6687 | 13491 | 3.9 | 52614.9 |
| **Carbohydrate** | 6811 | 6817 | 13628 | 3.9 | 53149.2 |
| **Fat** | 6232 | 6274 | 12506 | 5.2 | 65031.2 |
| **Protein** | 6659 | 6337 | 12996 | 3.9 | 50684.4 |

Group-level total energy intake was calculated by multiplying consumed food mass by the energy density (kcal/g) of the corresponding diet (Figure 1D).

**Supplementary Figure S1.** Weekly mean body-weight differences from control

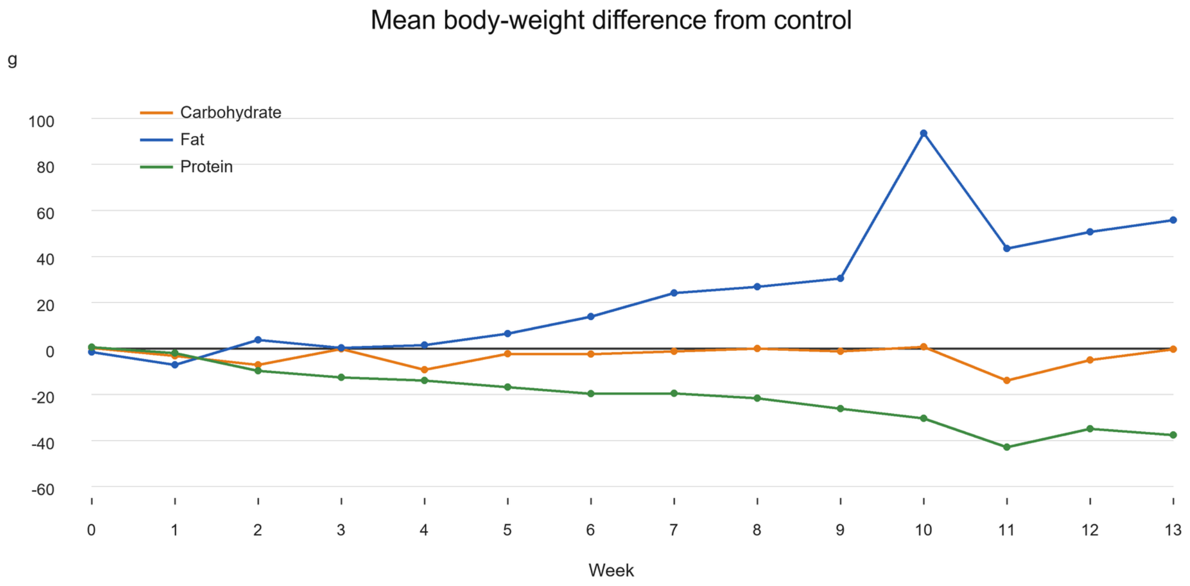


The graph shows differences in weekly group-mean body weights from the control group.
